# Excess Body Fat and Associated Factors in 7‐ to 10‐Year‐Old Schoolchildren in Florianópolis, Santa Catarina, Brazil: Trend Analysis of Four Cross‐Sectional Surveys, 2002–2019

**DOI:** 10.1002/ajhb.70141

**Published:** 2025-10-07

**Authors:** Francisca Maria Carvalho Nascimento, Patrícia de Fragas Hinnig, Mayara Luiza Vermohlem Garcia, Francisco de Assis Guedes de Vasconcelos, Cristine Garcia Gabriel

**Affiliations:** ^1^ Graduate Program in Nutrition, Department of Nutrition, Center for Health Sciences Federal University of Santa Catarina Trindade, Florianópolis Santa Catarina Brazil; ^2^ Department of Nutrition, School of Health Sciences University of Brasília Brasília Distrito Federal Brazil

**Keywords:** body fat distribution, children, schoolchildren, sociodemographic factors

## Abstract

**Objectives:**

This study aimed to assess the trend and factors associated with body fat percentage in 7‐ to 10‐year‐old schoolchildren in Florianópolis, Santa Catarina, Brazil, from 2002 to 2019.

**Methods:**

This is a trend analysis of four cross‐sectional surveys involving 6597 schoolchildren attending public and private schools. Body fat percentage (outcome variable) was calculated from triceps and subscapular skinfold thicknesses using Lohman equations and categorized into “no excess body fat” and “excess body fat.” Sociodemographic and socioeconomic characteristics were also investigated. Trends and associated factors were analyzed using multivariate logistic regression.

**Results:**

The prevalence of excess body fat was 22.6%, 25.5%, 37.5%, and 33.6% in 2002, 2007, 2013, and 2019, respectively, representing a 48.6% increase from 2002 to 2019. Schoolchildren assessed in 2013 (OR = 1.93) and 2019 (OR = 1.69), as well as those who were male (OR = 1.0) or older (9–10 years) (OR = 1.60), were more likely to have excess body fat.

**Conclusions:**

The increasing trends of body fat percentage and excess body fat in 7‐ to 10‐year‐old schoolchildren from 2002 to 2019 underscore the need for targeted strategies to address the growing rates of childhood obesity, with particular attention to high‐risk groups.

## 1 Introduction

In 2022, more than 390 million children aged 5–19 were classified as overweight or obese (NCD‐RisC 2024). For this age group, it is estimated that in 2035 half of Brazilian children will have this classification, corresponding to around 20.4 million (World Obesity Federation 2024). In South America, a meta‐analysis conducted between 2010 and 2020 identified that 28.3% of children and adolescents were overweight (overweight and obese), with a prevalence of 17.8% for overweight and 13.2% for obesity (Oliveira et al. 2022). This increase is concerning, as children with obesity are five times more likely to remain obese in adulthood than their non‐obese peers (Simmonds et al. 2016).

Most studies used body mass index (BMI) to assess the prevalence of overweight and obesity in schoolchildren (Tornquist et al. 2023; Simmonds et al. 2016). However, recognizing that BMI alone can underestimate or overestimate body fat, a study proposed the direct measurement of body fat, whenever possible, or the use of at least one additional anthropometric indicator, such as waist circumference, waist‐to‐hip ratio, or waist‐to‐height ratio, alongside BMI (Rubino et al. 2025). Consistent with these recommendations, another study supported the use of waist circumference as an indicator of central obesity in children and emphasized the importance of strengthening and expanding public actions and policies that promote a healthier and more active lifestyle (de Medeiros et al. 2025).

Evidence suggests that studies are increasingly using body fat percentage estimated from skinfold measurements to diagnose excess body fat in children. This method allows for a simpler and more direct interpretation of the results (Trang et al. 2019; Lohman 1986). In the past five years, countries such as Spain, Turkey, Bangladesh, and South Africa have conducted studies using skinfold measurements to assess body fat percentage, commonly adopting triceps and subscapular skinfold thicknesses as predictors of body fat (Calderón García et al. 2023; Gul Siraz et al. 2023; Kamruzzaman et al. 2021; Nkwana et al. 2019). However, a bibliographic search of the literature revealed few studies (Leal et al. 2015; Dereń et al. 2021; Wang et al. 2018) that explored the use of skinfold measurements to estimate body fat percentage in children, and even fewer that examined trends in body fat percentage over time.

In light of these considerations, this study aimed to use body fat percentage to identify trends and factors associated with excess body fat among 7‐ to 10‐year‐old schoolchildren in Florianópolis, SC, Brazil, between 2002 and 2019.

## 2 Materials and Methods

### 2.1 Study Design and Population

This is a cross‐sectional study using data from four survey waves conducted in 2002 (de Assis et al. 2005), 2007 (de Oliveira Bernardo and de Assis Guedes de Vasconcelos 2012), 2012/2013 (Motter et al. 2015; D'Avila et al. 2016), and 2018/2019 (Pereira et al. 2023). These surveys were part of the Obesity Prevalence Study in Children and Adolescents (EPOCA), performed by the Federal University of Santa Catarina (UFSC) in partnership with the Santa Catarina State Department of Education and the Florianópolis Municipal Department of Education.

The study population comprised children aged 7 to 10 years attending public or private schools in Florianópolis. Florianópolis is the capital of the state of Santa Catarina, a city located in southern Brazil, with a population of 537,211 (Instituto Brasileiro de Geografia e Estatística (IBGE) 2024) and a high municipal human development index (MHDI 0.847) (IBGE, 2024). The prevalence of overweight and obesity in this municipality has increased based on Body Mass Index data, particularly among children attending public schools. From 2002 to 2019, the prevalence of childhood obesity rose steadily, with no significant decline in thinness rates during this period (Spanholi et al. 2024).

The sampling process was similar between studies, although with some operational differences between surveys. Such differences are described in detail in published studies (de Oliveira Bernardo and de Assis Guedes de Vasconcelos 2012; D'Avila et al. 2016; de Medeiros et al. 2025; Motter et al. 2015; Pereira et al. 2023).

### 2.2 Data Collection

Data collection took place from September to November 2002 in the first survey (de Assis et al. 2005), from March to October 2007 in the second survey (de Oliveira Bernardo and de Assis Guedes de Vasconcelos 2012), from September 2012 to June 2013 in the third survey (Motter et al. 2015; D'Avila et al. 2016), and from November 2018 to December 2019 in the fourth and final survey (Pereira et al. 2023).

The four surveys included only schoolchildren who provided an informed consent form signed by parents/guardians and an assent form signed by themselves (last survey). Children with physical disabilities that precluded anthropometric evaluation, as well as those who provided incorrect data, were excluded from the study (de Assis et al. 2005; de Oliveira Bernardo and de Assis Guedes de Vasconcelos 2012; Motter et al. 2015; D'Avila et al. 2016; Pereira et al. 2023). Additionally, children aged 11–14 years and those lacking skinfold measurements were excluded.

For each survey, researchers were trained using pre‐determined protocols. Theoretical and practical workshops were conducted on measurement techniques, so as to standardize anthropometric evaluations (Lohman et al. 1988).

### 2.3 Study Variables

#### 2.3.1 Dependent Variable: Body Fat Percentage

The body fat percentage of participating schoolchildren was calculated from skinfold measurements, according to the method of Lohman (Lohman 1986, 1987). Triceps and subscapular skinfold measurements were determined according to procedures recommended in the literature (Lohman et al. 1988) and were collected using a TBW scientific caliper (Lange model, São Paulo, Brazil) with a scale ranging from 0 to 60 mm and a resolution of 1 mm. More detailed information on the standardization of collected skinfold measurements can be found in previously published studies (de Assis et al. 2005; de Oliveira Bernardo and de Assis Guedes de Vasconcelos 2012; Motter et al. 2015; D'Avila et al. 2016; Pereira et al. 2023).

Lohman equations were chosen because of their suitability to the studied age group, the availability of skinfold measurements, and the consideration of sex and age in model constants. Furthermore, various studies have applied these equations to investigate the nutritional status of children (de Assis et al. 2005; de Oliveira Bernardo and de Assis Guedes de Vasconcelos 2012; Motter et al. 2015; D'Avila et al. 2016; Pereira et al. 2023).

Schoolchildren with body fat percentages classified as very low, low, or normal were considered to have no excess body fat (boys, < 6.0%–20.0%; girls, < 12.0%–25.0%), whereas schoolchildren with high or very high body fat were considered to have excess body fat (boys, 20.1% to > 31.1%; girls, 25.1% to > 35.6%) (Lohman 1986, 1987).

#### 2.3.2 Independent Variables: Sociodemographic and Socioeconomic Characteristics

The independent variables were survey year (2002, 2007, 2012/2013, or 2018/2019), sex (male or female), age group (7–8 years or 9–10 years), school type (public or private), monthly household income (< 2 minimum wages or ≥ 2 minimum wages), and maternal education (< 8 years or ≥ 8 years). Minimum wage varied by survey year, as follows: R$200.00 in 2002, R$380.00 in 2007, R$622.00 in 2012, and R$998.00 in 2019. For maternal education, an 8‐year cutoff was used, as it represents the minimum years of study required to complete primary education. Maternal education was used as a proxy for socioeconomic status, as it reflects material, intellectual, and other family resources (Galobardes et al. 2006). Maternal education may impact child survival, including the mother's ability to: contribute to the family's income; command authority and make decisions in the family; make use of existing services; and provide child care (Barros et al. 2010). These data were collected from a printed questionnaire sent to parents/guardians together with the informed consent form (de Assis et al. 2005; de Oliveira Bernardo and de Assis Guedes de Vasconcelos 2012; Motter et al. 2015; D'Avila et al. 2016; Pereira et al. 2023).

### 2.4 Statistical Analysis

The data from the four EPOCA surveys were processed by a team of trained typists, who used a double data entry system (de Assis et al. 2005; de Oliveira Bernardo and de Assis Guedes de Vasconcelos 2012; Motter et al. 2015; D'Avila et al. 2016; Pereira et al. 2023). Manually collected data were entered independently and in duplicate into Epi Info version 3.3.2 (CDC, Atlanta, GA, United States). Analyses were performed using Stata version 13.1, and the svyset/svy command was applied to each survey database to account for the study design and sampling method.

The data were described separately for each survey using absolute and relative frequencies and 95% confidence intervals (CI). The median of dependent and independent variables was calculated as a measure of central tendency for comparison of body fat distributions between groups and identification of significant differences according to the 95% CI.

The four databases were merged into a single database to analyze the temporal trend of body fat percentage among schoolchildren. Initially, a crude analysis was conducted, in which each independent variable was tested individually against the outcome variable (body fat percentage). Subsequently, an adjusted analysis (Model 1) was performed, including all independent variables simultaneously in the regression model. Given the high proportion of missing data for monthly household income and maternal education, a sensitivity analysis was carried out (Model 2), in which missing values were treated as separate categories. This approach aimed to assess whether the lack of information influenced the results of the regression analysis. Logistic regression results are reported as odds ratios (ORs), with their respective 95% CIs and *p*‐values.

### 2.5 Ethical Aspects

The four surveys were approved by the Research Ethics Committee at the Federal University of Santa Catarina (protocols Nos. 037/2002, 028/2006, 120.341/2012, and 2.730.239/2018) (Pereira et al. 2023), in accordance with Resolution No. 196/96 (Brazil 1996) and Resolution No. 466/2012 of the National Health Council (Brazil 2013).

## 3 Results

This study included a total of 6597 schoolchildren aged 7–10 years, interviewed from 2002 to 2019. Table 1 describes the anthropometric, sociodemographic, and socioeconomic characteristics of the sample, stratified by year of the survey. In all years, most of the sample consisted of girls, mainly in the 9–10 age group. Most students attended public schools, and more than half had high family incomes. Maternal education varied more widely, but most mothers had at least 8 years of formal education (Table 1).

**TABLE 1:** Sample distribution of 7–10‐year‐old schoolchildren in Florianópolis, SC, Brazil, in 2002, 2007, 2013/2014, and 2018/2019, according to body fat status and sociodemographic and socioeconomic characteristics.

Variable	2002 (*n* = 2924)		2007 (*n* = 1232)		2012/2013 (*n* = 1520)		2018/2019 (*n* = 921)	
	*n*	95% CI (%)	*n*	95% CI (%)	*n*	95% CI (%)	*n*	95% CI (%)
Body fat status	(*n* = 2924)		(*n* = 1232)		(*n* = 1520)		(*n* = 921)	
No excess body fat	2254	**77.4 (70.0–83.2)** ^**a**^	923	**74.5 (71.4–77.2)**	947	62.5 (48.1–74.8)	609	**66.4 (63.2–69.4)**
Excess body fat	670	**22.6 (16.7–29.9)**	309	**25.5 (22.7–28.5)**	573	37.5 (25.1–51.8)	312	**33.6 (30.5–36.7)**
Sex	(*n* = 2924)		(*n* = 1232)		(*n* = 1520)		(*n* = 921)	
Male	1497	**50.9 (50.1–51.7)**	604	50.7 (47.7–53.7)	722	**45.0 (40.1–49.8)**	408	46.9 (43.5–50.2)
Female	1427	**49.1 (48.2–49.8)**	628	49.3 (46.2–52.2)	798	**55.0 (50.1–59.8)**	513	53.1 (49.7–56.4)
Age group	(*n* = 2924)		(*n* = 1232)		(*n* = 1520)		(*n* = 921)	
7–8 years	1437	49.3 (47.0–51.6)	565	45.7 (40.2–51.2)	777	56.5 (47.1–65.4)	407	**42.3 (38.6–46.0)**
9–10 years	1487	50.7 (48.3–52.9)	667	54.3 (48.7–59.7)	743	43.5 (34.5–52.8)	514	**57.7 (53.9–61.3)**
School type	(*n* = 2924)		(*n* = 1232)		(*n* = 1520)		(*n* = 921)	
Public	1900	**65.0 (63.2–66.6)**	950	63.5 (44.4–79.1)	994	**65.4 (62.9–67.7)**	570	**61.9 (58.7–64.9)**
Private	1024	**35.0 (33.3–36.7)**	282	36.5 (20.8–55.5)	526	**34.6 (32.2–37.0)**	351	**38.1 (35.0–41.2)**
Monthly household income^b^	(*n* = 2311)^c^		(*n* = 1043)^c^		(*n* = 1314)^c^		(*n* = 745)^c^	
< 2 minimum wages	625	28.2 (5.5–72.6)	252	**19.6 (14.0–26.6)**	283	20.1 (5.3–53.1)	224	**15.7 (4.4–43.1)**
≥ 2 minimum wages	1686	71.8 (27.3–94.4)	791	**80.4 (73.3–85.9)**	1031	79.9 (46.8–94.6)	521	**84.3 (56.8–95.5)**
Maternal education	(*n* = 1470)^c^		(*n* = 1185)^c^		(*n* = 1466)^c^		(*n* = 874)^c^	
≤ 8 years	396	28.25 (3.84–79.5)	529	**36.7 (26.7–47.8)**	371	21.9 (4.1–64.4)	177	**10.1 (2.5–32.0)**
≥ 8 years	1074	71.75 (20.4–96.1)	656	**63.3 (52.1–73.2)**	1095	78.1 (35.5–95.8)	697	**89.9 (67.9–97.4)**

Abbreviation: CI, confidence interval.

^a^ 95% CI values highlighted in bold indicate non‐overlapping intervals and suggest a statistically significant association.

^b^ Minimum wage in effect in the survey year.

^c^ The value of *n* is lower than the total number of observations in each survey due to missing data.

The prevalence of excess body fat among schoolchildren aged 7–10 years was 22.6%, 25.5%, 37.5%, and 33.6% in 2002, 2007, 2012/2013, and 2018/2019, respectively. This prevalence increased by 48.6% over the 17 years of evaluation. The variable increased between the first and last survey, and the highest overall percentage was recorded in 2012/2013 (Table 2).

**TABLE 2:** Description of the median body fat percentage overall and according to sociodemographic and socioeconomic characteristics among 7–10‐year‐old schoolchildren in Florianópolis, SC, Brazil, in 2002, 2007, 2013/2014, and 2018/2019.

Variable	2002 (*n* = 2924)		2007 (*n* = 1232)		2012/2013 (*n* = 1520)		2018/2019 (*n* = 921)	
	Median (%)	95% CI (%)	Median (%)	95% CI (%)	Median (%)	95% CI (%)	Median (%)	95% CI (%)
Body fat percentage	16.52	15.23–17.82	17.60	16.76–18.45	20.19	18.36–22.01	19.37	18.62–20.12
Sex								
Male	13.55	**11.96–15.13** ^**a**^	14.73	**13.96–15.50**	16.91	**14.48–19.35**	17.21	**16.34–18.09**
Female	18.82	**17.76–19.89**	19.73	**18.84–20.63**	22.49	**21.27–23.71**	21.05	**19.75–22.36**
Age group								
7–8 years	16.06	14.94–17.18	16.77	**15.66–17.88**	18.71	17.93–19.48	18.93	18.17–19.68
9–10 years	17.08	15.75–18.41	18.73	**18.16–19.31**	22.15	19.35–24.94	19.80	18.75–20.84
School type								
Public	15.82	**15.66–15.99**	17.16	15.98–18.34	18.91	**18.77–19.06**	19.90	19.56–20.25
Private	17.95	**17.89–18.00**	18.73	17.57–19.90	22.70	**22.48–22.92**	19.21	17.74–20.68
Monthly household income^b^								
< 2 minimum wages	15.45	**15.10–15.79**	17.15	16.02–18.28	18.91	17.32–20.51	19.21	18.05–20.37
≥ 2 minimum wages	17.38	**16.64–18.13**	18.01	16.97–19.05	20.2	18.22–22.17	19.79	18.95–20.63
Maternal education								
≤ 8 years	16.04	**15.90–16.17**	17.14	15.93–18.36	19.01	18.91–19.11	19.07	16.98–21.17
≥ 8 years	17.95	**17.19–18.70**	18.31	17.43–19.18	20.60	18.54–22.65	19.61	18.93–20.30

Abbreviation: CI, confidence interval.

^a^ 95% CI values highlighted in bold indicate non‐overlapping intervals and suggest a statistically significant association.

^b^ Minimum wage in effect in the survey year.

In all years of assessments, girls had a higher median body fat percentage than boys, with the largest difference recorded in the third survey (22.4% vs. 16.9%). Exceptionally, in the last survey, girls showed a small reduction compared to previous years, while boys maintained a progressive increase (Table 2).

Significant differences in body fat between age groups were only observed in the second survey, when students aged 9–10 years (18.7%, 95% CI = 18.16%–19.31%) had higher median body fat than students aged 7–8 years (16.7%, 95% CI = 15.66%–17.88%) (Table 2).

In 2002 and 2012/2013, private school students had significantly higher average body fat levels than public school students. Specifically, in 2002, children of mothers with higher education (more than 8 years) had a higher median body fat percentage than those whose mothers had less than 8 years of schooling. Regarding household income, there was a significant increase in body fat percentage over the years, with this variable being statistically significant in 2002.

In the adjusted analysis of the main model (Model 1), schoolchildren assessed in 2012/2013 and 2018/2019 had 93% and 69% higher odds, respectively, of having excess body fat than children evaluated in 2002 (Table 3). Girls were less likely (OR = 0.80, 95% CI = 0.70–0.92) to have excess body fat than boys. Older students (9–10 years old) were 60% more likely (OR = 1.60) to have excess body fat than younger students (7–8 years old) (Table 3). There was no association of excess body fat with school type, household income, or maternal education in the main analysis (Table 3).

**TABLE 3:** Crude and adjusted regression analysis for excess body fat among 7–10‐year‐old schoolchildren in Florianópolis, Brazil, in 2002, 2007, 2012/2013, and 2018/2019, according to sociodemographic and socioeconomic factors.

Variable	Crude OR^a^			Adjusted OR (Model 1)^a^			Crude OR^b^			Adjusted OR (Model 2)^b^		
	OR	95% CI (%)	*p*	OR	95% CI (%)	*p*	OR	95% CI (%)	*p*	OR	95% CI (%)	*p*
Excess body fat												
Survey year	**(*n* = 6597)**						**(*n* = 6597)**			**(*n* = 6597)**		
2002	1			1			1			1		
2007	1.12	0.96–1.31	0.133	1.06	0.88–1.29	0.498	1.12	0.96–1.31	0.133	1.06	0.89–1.27	0.459
2012/2013	2.03	1.77–2.32	**0.000**	1.93	1.64–2.29	**0.000**	2.03	1.77–2.32	**0.000**	1.92	1.65–2.24	**0.000**
2018/2019	1.72	1.46–2.02	**0.000**	1.69	1.39–2.06	**0.000**	1.72	1.46–2.02	**0.000**	1.60	1.34–1.91	** 0.000**
Sex	**(*n* = 6597)**						***n* = 6597**					
Male	1			1			1			1		
Female	0.88	0.78–0.97	**0.018**	0.80	0.70–0.92	**0.001**	0.88	0.78–0.97	**0.018**	0.85	0.76–0.95	**0.004**
Age group	**(*n* = 6597)**						***n* = 6597**					
7–8 years	1			1			1			1		
9–10 years	1.52	1.37–1.70	**0.000**	1.60	1.40–1.83	**0.000**	1.52	1.37–1.70	**0.000**	1.55	1.39–1.73	**0.000**
School type	**(*n* = 6597)**						**(*n* = 6597)**					
Public	1			1			1			1		
Private	1.25	1.12–1.40	**0.000**	1.11	0.94–1.30	0.187	1.25	1.12–1.40	**0.000**	1.18	1.04–1.35	**0.001**
Monthly household income^c^	**(*n* = 5413)** ^**d**^						**(*n* = 6597)**					
< 2 minimum wages	1			1			1			1		
≥ 2 minimum wages	1.26	1.09–1.44	**0.001**	1.07	0.90–1.27	0.423	1.26	1.09–1.44	**0.001**	1.12	0.96–1.30	0.133
Missing data^e^	—	—	—	—	—	—	0.95	0.79–1.13	0.594	0.93	0.76–1.13	0.494
Maternal education	**(*n* = 4995)** ^**d**^						**(*n* = 6597)**					
≤ 8 years	1			1			1			1		
≥ 8 years	1.22	1.06–1.39	**0.004**	1.11	0.94–1.32	0.189	1.22	1.06–1.39	**0.004**	1.05	0.90–1.22	0.520
Missing data^e^	—	—	—	—	—	—	0.72	0.61–0.85	**0.000**	0.89	0.73–1.09	0.286

*Note:* Values of *p* < 0.05 indicate statistically significant associations. Bold values represent *p*‐values (< 0.05) that were considered statistically significant.

Abbreviations: CI, confidence interval; OR, odds ratio.

^a^ Analysis performed by excluding missing data.

^**b**^ Analysis conducted by imputing values for missing data.

^c^ Minimum wage in effect in the survey year.

^d^ The value of *n* is lower than the total number of observations in each survey due to missing data.

^e^ Values assigned to missing data.

Sensitivity analysis for missing data on maternal education and household income revealed a similar trend of increased odds of having excess body fat in 2012/2013 and 2018/2019 as compared with 2002. Different results were observed for school type, in that there was a positive association between being enrolled in a private school and having excess body fat (OR = 1.18, 95% CI = 0.94–1.30) (Table 3).

## 4 Discussion

The main findings of the study indicate an increasing trend in body fat percentage among schoolchildren aged 7–10 years, rising from 22.6% in 2002 to 33.6% in 2019.

Few recent studies used skinfold measurements to measure body fat percentage in children aged 7–10 years, hindering comparison of the results. The current study is relevant, given its large sample size and extended analysis period, ranging over 20 years. Furthermore, it is one of the few investigations using skinfold measurements to analyze body fat among schoolchildren aged 7–10 years.

A study conducted in Switzerland (Herter‐Aeberli et al. 2019) in 2002, 2007, and 2017/2018 included schoolchildren aged 6–13 years and used triceps, biceps, subscapular, and suprailiac skinfold measurements to determine body fat percentage. The authors reported body fat percentages of 18.2%, 19.3%, and 17.1% in 2002, 2012, and 2017/2018, respectively (Herter‐Aeberli et al. 2019), demonstrating a reduction over time, a result opposite to that observed here.

In China, a study of children and adolescents aged 7–18 (Wang et al. 2018) assessed the prevalence of elevated skin folds over 19 years (1995–2014). A significant increase in prevalence was observed, from 10.3% to 33.9% among boys and from 13.4% to 29.3% among girls, suggesting an increase in body fat percentage. As such, these findings support those of the current study, conducted over a similar period (Wang et al. 2018).

In Brazil, a study conducted in Lavras, Minas Gerais State, with 90 schoolchildren aged 8–10 years attending public schools, reported body fat percentages of 23.5% (22.1% in boys and 24.2% in girls) based on triceps and subscapular skinfold measurements (Botelho‐Santos et al. 2017). In Porto Alegre, Rio Grande do Sul State, a mean body fat percentage of 20.4% was found among 439 participants aged 5–18 years from 2015 to 2018 (Forte et al. 2021). It should be noted that both studies (Botelho‐Santos et al. 2017; Forte et al. 2021) used the same equation as the one adopted in the current study to calculate body fat percentage.

Here, survey year, sex, and age were found to significantly influence body fat percentage, as evidenced by logistic regression. This finding agrees with those of a study conducted in Ilhabela, São Paulo State, in which girls had higher adiposity than boys (Lopes et al. 2022). Similarly, girls had higher triceps and subscapular skinfold thicknesses than boys in a Colombian study involving schoolchildren aged 9–17 years (Ramírez‐Vélez et al. 2016). However, in the current study, logistic regression showed that girls exhibited lower odds of having excess body fat (OR = 0.80), suggesting a protective effect of the female sex.

Thus, although girls had higher median values of body fat percentage, they were not necessarily classified as having excess body fat according to sex and age. An explanation for this result may be the stage of sexual maturation of participants. Differences in body fat accumulation between the sexes appear at specific stages of development (Staiano and Katzmarzyk 2012). Studies indicate that children and adolescents of both sexes undergoing early puberty may have greater central adiposity (Adami et al. 2020). It has been shown that prepubescent boys have greater fat mass than pubescent boys, while prepubescent girls have greater muscle mass, especially in the central region (Adami et al. 2020; de Medeiros et al. 2025).

It is emphasized that during and after puberty, girls tend to accumulate more total and subcutaneous body fat, concentrating greater volume in areas such as the hips and lower limbs. In contrast, boys in puberty and post‐puberty accumulate more fat in the abdominal region, especially in the visceral form (Staiano and Katzmarzyk 2012). In addition, in girls, being overweight or obese is associated with earlier sexual maturation, including earlier menarche (Matsuo et al. 2022). These relationships are concerning, as early puberty is associated with an increased risk of metabolic diseases such as obesity, diabetes, and cardiovascular disease (Sun et al. 2024).

Overall, median body fat percentage did not differ significantly between age groups (7–8 years and 9–10 years). However, in the logistic regression, students aged 9–10 years had greater odds (OR = 1.60) of having excess body fat. It should be noted that significant increases in the risk of excess body fat among older boys in this study may be related to the peak of puberty, when greater fat accumulation occurs, unlike girls, who show stabilization in this accumulation (Adami et al. 2020). A similar gender difference was observed in the United States (US) study with a representative sample of children and adolescents between 5 and 18 years of age, in which skinfold measurements indicated that body fat percentage increased progressively in girls during childhood and adolescence. On the other hand, in boys, body fat percentage peaked at approximately 11 years of age, followed by a gradual reduction (Laurson et al. 2011).

Although most of the sample was from public schools, private school students had higher median body fat percentages in all assessments, a finding that was reinforced by regression analysis (Model 2). de Lima Mendes Ramos et al. (2013) analyzed the body fat percentage of schoolchildren using triceps and subscapular skinfold measurements. The authors found that private school students exhibited higher odds (OR = 1.3) of having high body fat compared with public school students (de Lima Mendes Ramos et al. 2013). According to Leal et al. (2015), who also analyzed data from the EPOCA survey, enrollment in private or public schools in Florianópolis, Santa Catarina State, was highly dependent on monthly household income in 2002 and 2007 (Leal et al. 2015).

As a limitation, the last survey had a low participation rate. Many students did not return the informed consent and assent forms. Furthermore, the questionnaire sent to parents/guardians was quite long, which may have contributed to defaults (Pereira et al. 2023). In all surveys, various students did not provide information on monthly household income and maternal education. Obtaining accurate answers about income is a recurrent challenge in research in view of labor instability, varied incomes, or participants' reluctance to report these data (Pérez et al. 2022). The lack of this information can introduce a selection bias in the sample, making it necessary to adopt alternative methods, such as data imputation, to preserve the information and mitigate potential biases. Another option is sensitivity analysis to take into account missing data, as was performed in the current study (Alves and Soares 2009). Throughout the statistical analysis and discussion, it was observed that not including the sexual maturation variable in the investigation may have been a limitation since, as discussed, it is a biological characteristic that interferes with the outcome of the research. A strength of this research was the standardization of procedures across all surveys, including training of the data collection team, consistent measurement protocols, double data entry verification, and outlier analysis.

## 5 Conclusion

There was a significant increasing trend in body fat percentage among schoolchildren aged 7–10 years in Florianópolis from 2002 to 2019. The following factors were associated with excess body fat: survey year (2012/2013 and 2018/2019), sex (boys), older age (9–10 years), and school type (private school). The results of this study may inform clinical practice by supporting more targeted interventions for children's health. Results such as those from this study can contribute to strategies for evaluating and improving public policies aimed at schoolchildren, integrating these data into existing public health programs and policies. Within the scope of the National School Feeding Program (PNAE), the findings may encourage the implementation of actions such as ongoing training for school meal staff and nutritionists in the municipality of Florianópolis, in addition to encouraging the participation of the school community in promoting healthy eating. Complementarily, through the Health at School Program (PSE), lectures and campaigns on healthy eating and encouraging physical activity can be offered, as well as reinforcing the importance of monitoring health indicators. Additionally, initiatives have been developed in both public and private schools to restrict the provision of foods high in sugar, fried foods, or ultra‐processed foods. The results of this study can help inform inspection efforts and the implementation of measures aimed at improving children's quality of life and health indicators, in addition to encouraging school cafeterias across the country to be regulated for this purpose.

## Author Contributions

Francisca Maria Carvalho Nascimento: responsible for conducting the literature review, organizing the data, performing statistical analysis, and preparing the manuscript. Patrícia de Fragas Hinnig: responsible for coordinating the 2018 survey, guiding statistical analyses, and reviewing the manuscript. Mayara Luiza Vermohlem Garcia: responsible for assisting in the statistical analysis and reviewing the manuscript. Francisco de Assis Guedes de Vasconcelos: responsible for coordinating and planning the original studies, supervising, and reviewing the manuscript. Cristine Garcia Gabriel: responsible for supervising and reviewing the manuscript.

## Conflicts of Interest

The authors declare no conflicts of interest.

## Data Availability

The data that support the findings of this study are available on request from the corresponding author. The data are not publicly available due to privacy or ethical restrictions.
